# Deep Venous Thrombosis of the Leg, Associated with Agenesis of the Infrarenal Inferior Vena Cava and Hypoplastic Left Kidney (KILT Syndrome) in a 14-Year-Old Child

**DOI:** 10.1155/2015/864047

**Published:** 2015-01-05

**Authors:** Sakshi Bami, Yarelis Vazquez, Valeriy Chorny, Rachelle Goldfisher, John Amodio

**Affiliations:** ^1^Department of Pediatrics, SUNY Downstate Medical Center, 450 Clarkson Avenue, Brooklyn, NY 11203, USA; ^2^Department of Radiology, SUNY Downstate Medical Center, 450 Clarkson Avenue, Brooklyn, NY 11203, USA

## Abstract

Agenesis of the inferior vena cava (IVC) is a rare anomaly which can be identified as incidental finding or can be associated with iliofemoral vein thrombosis. IVC agenesis has a known association with renal anomalies which are mainly confined to the right kidney. We describe a case of a 14-year-old male who presented with left leg swelling and pain. Ultrasonography confirmed the presence of left leg deep vein thrombosis (DVT). No underlying hematologic risk factors were identified. A CT scan was obtained which demonstrated absent infrarenal IVC and extensive thrombosis in the left deep venous system and development of collateral venous flow into the azygous/hemiazygous system, with extension of thrombus into paraspinal collaterals. An additional finding in the patient was an atrophic left kidney and stenosis of an accessory left renal artery. Agenesis of the IVC should be considered in a young patient presenting with lower extremity DVT, especially in patients with no risk factors for thrombosis. As agenesis of the IVC cannot be corrected, one should be aware that there is a lifelong risk of lower extremity DVT.

## 1. Introduction

Deep vein thrombosis occurs with a prevalence of 1 in 1000 [1]. It is seen less in younger population with an estimated incidence of 1 in 10,000. Hematologic risk factors associated with deep vein thrombosis can be congenital and/or acquired. In up to 80% of the cases of DVT, one or more risk factors can be identified [1].

Anomalies of the inferior vena cava (IVC) are an independent risk factor for DVT. These anomalies are found in 0.3–0.5% of the general population and in 0.6–2% of patients with cardiovascular defects. The most common anomalies of IVC are double IVC, left sided IVC, IVC agenesis or absence, and retroaortic left renal vein [2]. In young patients with DVT, there is an estimated higher rate of anomalies of IVC than in the general population, namely, 5% as compared to 0.5% expected [3, 4].

We describe a case of a 14-year-old male who presented with left leg swelling and pain. Ultrasonography confirmed the presence of left leg deep vein thrombosis (DVT). No underlying hematologic risk factors were identified. CT scan was obtained which demonstrated absent infrarenal IVC and extensive thrombosis in the left deep venous system and development of collateral venous flow into the azygous/hemiazygous system, with extension of thrombus into paraspinal collaterals. An additional finding in the patient was an atrophic left kidney and stenosis of an accessory left renal artery.

## 2. Case Report

A 14-year-old male presented to the emergency department with complaint of left lower extremity pain for 5 days. The pain was localized to the left thigh, worsening over time despite analgesic intake. Patient also complained of swelling of the thigh, difficulty in ambulation for 2 days, and numbness for 1 day. There was no history of trauma, recent surgery, medication use, or prolonged immobilization. There was no family history of clotting or bleeding disorder or venous thromboembolism. He denies any history of smoking or illicit drug use.

Patient is a known case of type 1 diabetes mellitus diagnosed at age of 10 years, currently on insulin pump. He was diagnosed with hypertension at age 9 and is on enalapril. He was born in Jamaica, via normal spontaneous vaginal delivery at term and had shoulder dystocia at birth for which he stayed in the hospital for 10 days. A sling was applied and no other intervention was done. Patient's mother denied any other complications at birth.

On examination he was noted to have marked asymmetry between the two lower extremities. There was tense swelling of the left posterior thigh and the left calf, which was tender to palpation. No erythema, warmth, varicose veins, or ulcers were present. Peripheral pulses were palpable and equal bilaterally with normal neurological exam.

**Figure 1:**
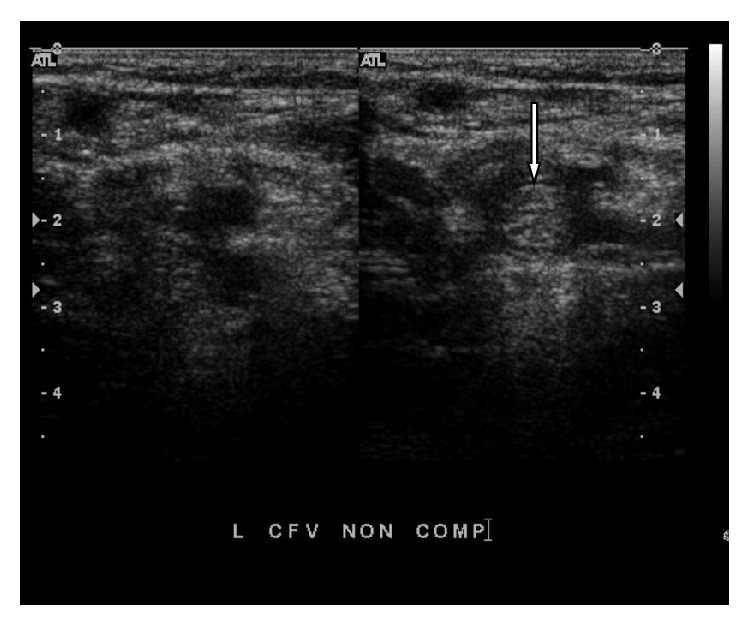
Grayscale ultrasound transverse image demonstrating a thrombus on the left common femoral vein. White arrow is pointing to the intraluminal echogenic material and noncompressibility, findings compatible with deep vein thrombosis.

His initial laboratory results in the emergency room showed normal complete blood count, basic metabolic panel, prothrombin time, and activated partial thromboplastin time. A lower extremity ultrasound showed the left common femoral, left superficial femoral, and left popliteal vein were noncompressible and demonstrated no vascular flow, with intraluminal echogenic thrombus suggestive of deep vein thrombosis of the left lower extremity (Figure 1).

He was admitted to the pediatric floor and started on low molecular weight (LMW) heparin and warfarin after hematology consultation. His chest X-ray was normal. A thrombophilia workup was done which showed no prothrombin gene mutation, normal levels of Factor V Leiden, antithrombin III, and protein S. Protein C was low 51.9 (normal 55–123 units IU/dL). Low protein C in the setting of a large DVT was attributed to consumption of coagulation factors. LDH, uric acid, and homocysteine level were normal. Anticardiolipin and lupus anticoagulant were normal.

**Figure 2:**
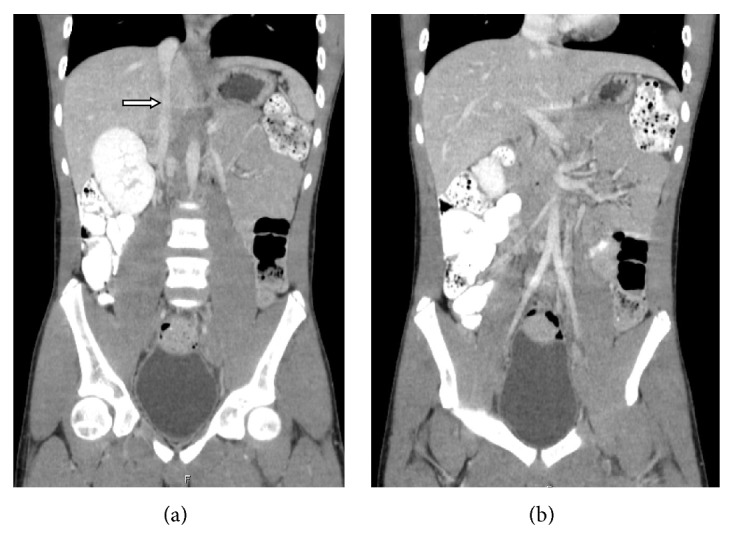
Contrast enhanced abdominopelvic CT scan. Two contiguous coronal reformations showing patent suprarenal inferior vena cava (white arrow). Note absence of the infrarenal inferior vena cava in the right image.

**Figure 3:**
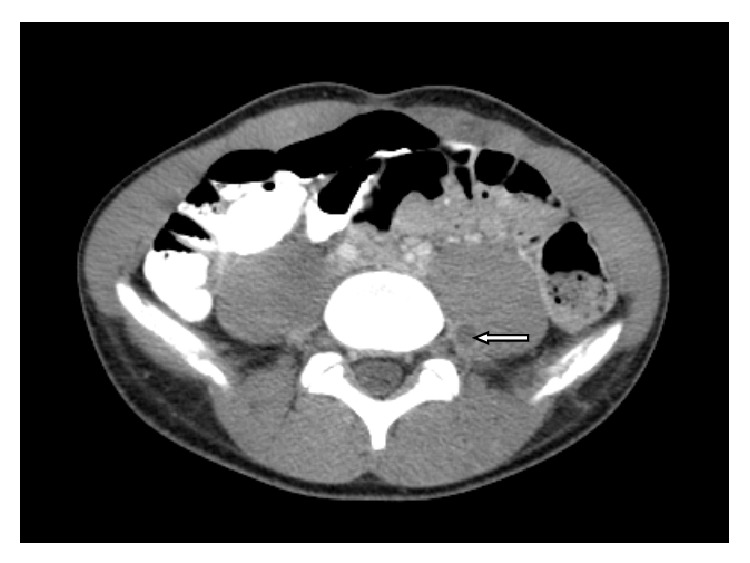
There is thrombus noted within a paraspinal collateral vein (white arrow).

**Figure 4:**
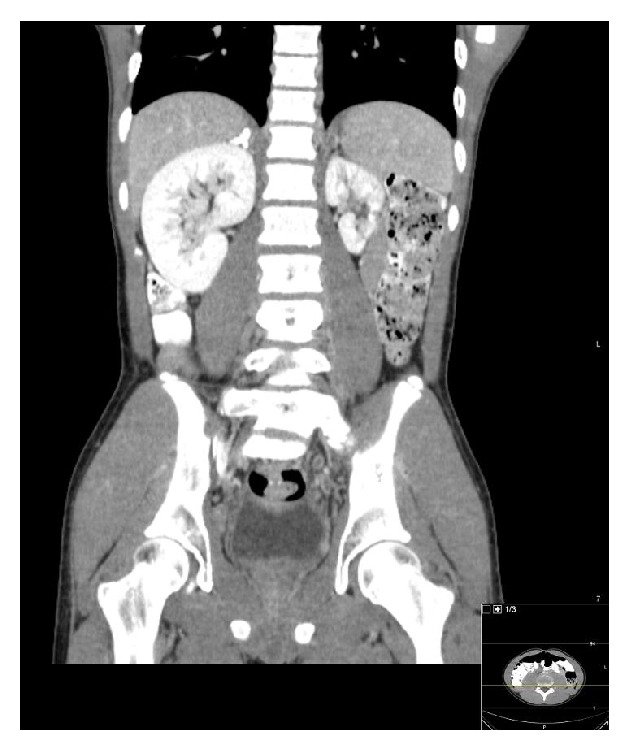
Contrast enhanced abdominopelvic CT scan. Coronal reformation demonstrating left renal hypoplasia with compensatory right renal hypertrophy. The right adrenal gland is heavily calcified.

A CT of the abdomen and pelvis was done to determine the extent of the thrombosis in the pelvis. The CT showed the suprarenal IVC and the hepatic segments of the IVC were patent. There was absence of infrarenal IVC (Figure 2). There was an anomalous course of the external iliac veins communicating with lumbar veins. There was heterogeneous material within the left common femoral vein and left external iliac vein and hypodensity within the left lumbar vein consistent with thrombus (Figure 3). There were prominent azygous and hemiazygous veins. The left kidney was small in size and there was compensatory hypertrophy of the right kidney (Figure 4). The renal veins were not thrombosed and the origin of the left renal vein was normal in caliber. There was calcification of the right adrenal gland noted consistent with prior adrenal hemorrhage, the etiology of which could not be ascertained.

**Figure 5:**
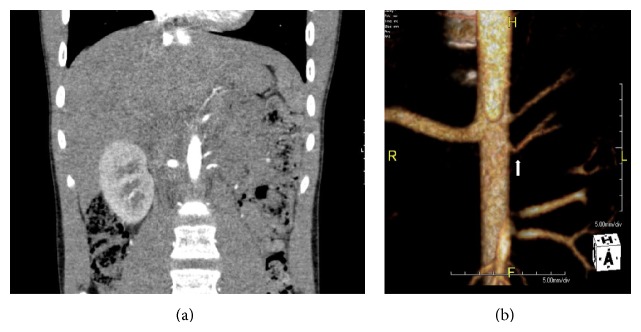
(a) Abdominal CTA coronal reformation and (b) three-dimensional reconstruction showing the left kidney supplied by two hypoplastic renal arteries originating from the aorta. The origin of the inferior left renal artery has a focal area of stenosis proximally, depicted by the white arrow.

On review of patients past medical records it was noted that as a workup of hypertension he had a CT angiogram done which demonstrated atrophic left kidney supplied by two hypoplastic renal arteries arising from the abdominal aorta. The origin of the more inferior renal artery had a short segment of stenosis (Figure 5). The right kidney was normal. A DMSA renal scan done subsequently demonstrated left renal uptake of approximately 13% and right renal uptake of approximately 87%.

The patient was continued on low molecular weight (LMW) heparin until his international normalized ratio (INR) reached more than 2. His pain and stiffness improved and he was discharged on oral warfarin therapy. Patient and mother were made aware that he may need lifelong anticoagulation therapy. In view of the fact that the patient had venous and arterial anomalies, prior to discharge the patient received a brain MRA/MRV to look for any other vascular anomalies, which were normal. A genetic evaluation was also normal. The patient is being followed by hematology team for venous thrombosis as an outpatient and is on oral warfarin therapy with therapeutic INR.

## 3. Discussion

The embryogenesis of IVC is complex. The normal IVC is composed of four segments: hepatic, suprarenal, renal, and infrarenal. The hepatic segment is derived from the vitelline vein. The right subcardinal vein develops into the suprarenal segment by formation of the subcardinal-hepatic anastomosis. The renal segment develops from the right suprasubcardinal and postsubcardinal anastomoses. It is generally accepted that the infrarenal segment derives from the right supracardinal vein. In the thoracic region, the supracardinal veins give rise to the azygos and hemiazygos veins. In the abdomen, the postcardinal veins are progressively replaced by the subcardinal and supracardinal veins but persist in the pelvis as the common iliac veins [5]. Some authors propose that absence of the IVC may be a result of perinatal thrombosis or intrauterine thrombosis, with obliteration and subsequent resorbtion [6]. Without normal development of the infrarenal IVC, the iliofemoral veins drain into the azygous and hemiazygous veins via anterior paravertebral collaterals. As all of the collateral vessels are much smaller in caliber with respect to the normal IVC, it is understandable that such collateral pathways may lead to chronic venous stasis and thrombosis of the lower extremity.

There are several variations in the anatomy of the IVC [5]. These include, among several, the following.

1. Left IVC results from regression of the right supracardinal vein with persistence of the left supracardinal vein. The prevalence is 0.2%–0.5%.
2. Duplication of the IVC results from persistence of both supracardinal veins. The prevalence is 0.2%–3%.
3. Azygos continuation of the IVC has also been termed absence of the hepatic segment of the IVC with azygos continuation. The prevalence is 0.6%. Agenesis of hepatic segment of IVC with azygous continuation is well defined entity in literature and is often seen in conjunction with other congenital anomalies, such the heterotaxy syndromes, specifically polysplenia.
4. Circumaortic left renal vein results from persistence of the dorsal limb of the embryonic left renal vein and of the dorsal arch of the renal collar (intersupracardinal anastomosis). The prevalence may be as high as 8.7%.
5. Retroaortic left renal vein results from persistence of the dorsal arch of the renal collar.

Agenesis of the infrarenal IVC is a rare anomaly, and the other associated anomaly usually involves the kidneys. The renal anomalies associated with absent IVC are mostly found to be confined to the right kidney [7]. The anomalies noted are right renal hypoplasia, aplasia, or agenesis. These anomalies are not an incidental finding and can be explained by abnormal venous embryogenesis. If there is anomalous development of the IVC which impairs venous drainage of the right metanephros, there may be aplasia or hypoplasia of the right kidney. Since the venous drainage of the left metanephros is via the gonadal vein and lumbar perforators, it is much less common to have left renal anomalies associated with IVC anomalies [8]. However, there are a few case reports in literature of absent IVC associated with left renal anomalies. Iqbal and Nagaraju [9] described a case of 54-year-old man with agenesis of the IVC and left kidney agenesis. van Veen et al. [10] described a case of 12-year-old girl who presented with bilateral DVT and agenesis of IVC with hypoplastic left kidney. Lawless and Dangleben [11] described agenesis of the infrarenal IVC associated with left hypoplastic kidney, in a 50-year-old male. van Veen et al. [10] have proposed that the association of DVT with agenesis of the IVC and associated renal anomalies be termed “KILT” syndrome (kidney anomaly, inferior vena cava anomaly, and leg thrombosis).

The cause of the left renal hypoplasia in the case we report may be twofold; the hypoplastic arteries associated with the left kidney suggest a congenital etiology, but it is uncertain if there was a venous anomaly based on absence of the infrarenal IVC. Additionally, the renal artery stenosis associated with the most inferior renal artery may have added to the hypoplasia of the kidney and subsequently the patient's hypertension.

Patients with IVC agenesis can present with a varied clinical picture. Some can be asymptomatic and absence of the IVC is found as an incidental finding; others can present with venous thrombosis and its consequences as in our case. DVT associated with IVC agenesis usually affects young population and is often bilateral [3, 12]. It commonly involves the iliac veins and is often recurrent [13]. Although uncommonly reported, patients may also present with pulmonary embolism.

The renal veins were not thrombosed and the origin of the left renal vein was normal in caliber. Additionally, there was no evidence of a “nutcracker” phenomenon. In the nutcracker syndrome the left renal vein is compressed between the superior mesenteric artery and the aorta. Symptoms vary in this anomaly; some children are asymptomatic while others may present with hematuria, pain, and varicoceles [14].

There has not been a clear consensus in the literature about the management of patients with agenesis of the IVC and venous thrombosis. Most of the patients described have been treated with anticoagulation and elastic stockings. Few have required surgical intervention for relief of symptoms. The length of treatment with anticoagulation has also not been well described in literature. Unlike acquired risk factors which may be correctable, patients with the risk factor of absent infrarenal IVC may be at a lifelong risk for thromboembolism. More followup studies on these patients to determine their recurrence of symptoms, length of treatment, alternate treatment options, and prognosis are needed.
